# Artificial Diamonds

**Published:** 1876-11

**Authors:** 


ARTIFICIAL DIAMONDS.—Many pastes have been devised for the preparation of these beautiful stones. None have been able in their products to equal the natural diamond in hardness and brilliancy; but nevertheless, to an unpracticed eye, the imitation is so perfect that the difference cannot be distinguished. The following formula for making a diamond paste is said to be one that gives the most satisfactory result. It is called Loysel's paste:

| | |
|---|---|
| Take pure silica, | 100 parts. |
| Red oxide of lead, | 150 " |
| Potash, calcined, | 30 " |
| Borax, calcined, | 10 " |
| Arsenious acid, | 1 " |

This produces a paste which has great brilliancy and refractive and dispersive powers, and also a specific gravity similar to that of the oriental diamond. It fuses at a moderate heat, and acquires the greatest brilliancy when remelted and kept for two or three days in a fused state, in order to expel the superabundant alkali and perfect the refining. This paste is used not only to produce factitious diamonds, but, besides, other factitious gems, of which it forms the basis.—*Druggists' Circular.*

